# Evaluation of a Strength-Based Resilience Program on Resilience, Perceived Stress, and Depressive Symptoms Among Secondary School Students in a Thai Private School: A Single-Group Pre–Post–Follow-Up Mixed-Methods Study

**DOI:** 10.3390/healthcare14152337

**Published:** 2026-08-01

**Authors:** Siriphorn Na nakorn, Wilai Napa, Suchanart Inwanna

**Affiliations:** Ramathibodi School of Nursing, Faculty of Medicine Ramathibodi Hospital, Mahidol University, Bangkok 10400, Thailand; siriphorn.nan@mahidol.ac.th (S.N.n.); wilai.nap@mahidol.ac.th (W.N.)

**Keywords:** resilience, psychological, adolescent, mental health, stress, psychological, depression, school health services, Thailand, mixed methods

## Abstract

**Highlights:**

**What are the main findings?**

- A six-session school-based resilience program (CMCL) was associated with reductions in perceived stress and depressive symptoms, and significant gains in resilience, maintained at one-month follow-up among male students at a Thai private secondary school.
- Qualitative analysis identified three themes—self-understanding, stress management, and understanding others—offering an interpretive account of how the program may produce change beyond what outcome measures capture.

**What are the implications of the main findings?**

- The CMCL program appears feasible, timetable-compatible, and culturally appropriate as a school-based mental health intervention; controlled studies with larger and more diverse samples are needed before broader implementation in Thai secondary schools can be recommended.
- Schools represent a critical setting for preventive resilience programming; integrating structured programs like CMCL may help address rising adolescent mental health problems before they reach clinical severity.

**Abstract:**

**Background/Objectives**: Adolescent mental health is a growing public health concern in Thailand, with high rates of stress and depressive symptoms among secondary school students. This study aimed to (1) quantitatively examine changes in resilience, perceived stress, and depressive symptoms associated with participation in the Changing Minds, Changing Lives (CMCL) strength-based resilience program and (2) qualitatively explore participants’ experiences of the program, at a private single-sex secondary school in Bangkok, Thailand. **Methods**: A quasi-experimental, single-group, pre–post–follow-up mixed-methods design, without a control group, was employed. Twenty-six students aged 15–18 years, enrolled in a single-sex boys’ secondary school, were recruited using convenience sampling. The Thai Connor–Davidson Resilience Scale (Thai CD-RISC), Thai Perceived Stress Scale (T-PSS-10), and Thai Patient Health Questionnaire-9 (PHQ-9) were administered at baseline (T_1_), immediately post-intervention (T_2_), and one-month follow-up (T_3_). The CMCL program comprised six sessions delivered over three weeks. Repeated measures ANOVA with Bonferroni post hoc correction was used for quantitative analysis. Qualitative data from reflective writings and final presentations were analyzed using conventional content analysis. **Results**: All outcomes showed significant within-group change over time. Resilience increased from pre-intervention (T_1_: *M* = 64.00) to post-intervention (T_2_: *M* = 76.46) and one-month follow-up (T_3_: *M* = 78.19; *F*_(2,50)_ = 75.63, *p* < 0.001, η^2^ = 0.752). Perceived stress decreased from pre-intervention (T_1_: *M* = 20.62) to post-intervention (T_2_: *M* = 13.04) and one-month follow-up (T_3_: *M* = 9.62; *F*_(1.567,39.17)_ = 105.03, *p* < 0.001, η^2^ = 0.808). Depressive symptoms also declined from pre-intervention (T_1_: *M* = 7.27) to post-intervention (T_2_: *M* = 5.81) and one-month follow-up (T_3_: *M* = 4.88; *F*_(2,50)_ = 14.74, *p* < 0.001, η^2^ = 0.371). Qualitative findings supported these results, highlighting three themes: Self-Understanding, Stress Management, and Understanding Others. **Conclusions:** Participation in the CMCL strength-based resilience program was associated with increased resilience and reduced perceived stress and depressive symptoms among Thai secondary school students, with changes maintained at one-month follow-up. Given the single-group design and small sample, these findings are preliminary. The program appears feasible and promising as a school-based preventive mental health intervention for adolescents in Thailand, and warrants evaluation in controlled studies with larger and more diverse samples.

## 1. Introduction

Adolescent mental health is a growing global and national public health priority. A meta-analysis of studies conducted during the COVID-19 pandemic reported that approximately 25% of teenagers worldwide exhibit clinically significant depressive symptoms, while anxiety affects approximately 20% [1]. In Thailand, national survey data indicate that approximately 37.8% of adolescents are at risk for depression, and around 22% have reported suicidal ideation [2]. Urban secondary school students face particularly intense academic pressures; approximately 26% of Thai upper secondary school students report significant educational stress [3]. Chronic stress and depression during adolescence are associated with mental health disorders, impaired academic performance, and adverse long-term well-being outcomes, highlighting the need for effective protective strategies.

Resilience—the capacity to adapt positively to adversity and recover from stress—is recognized as a key protective factor that may buffer the effects of stress and reduce the risk of depression [4]. Resilience is conceptualized not as a fixed trait but as a dynamic, learnable capacity that develops through the interaction of individual and environmental resources. It influences individual outcomes through several interrelated mechanisms. Cognitively, resilient individuals tend to appraise stressors as challenges rather than threats and use cognitive reappraisal to reinterpret adverse experiences in more adaptive terms, dampening physiological and emotional stress responses [5]. Emotionally, resilience involves regulatory skills that limit the intensity and duration of negative affect, thereby reducing vulnerability to depressive symptoms. Behaviorally, resilient individuals draw actively on personal strengths and problem-solving strategies rather than avoidance. Socially, resilience is sustained by supportive relationships that provide emotional resources and buffer isolation. Because these appraisal, regulatory, and relational capacities are modifiable, structured programs can strengthen resilience, and adolescence—a period of heightened neuroplasticity, identity formation, and rising stress exposure—represents an especially opportune developmental window for such intervention.

Fostering resilience through school-based programs offers a scalable, non-clinical preventive approach to promoting adolescent mental health. Strength-based resilience programs, rooted in positive psychology, emphasize the identification and development of individual strengths and assets rather than focusing solely on deficits or pathology [6].

Evidence from intervention studies supports the effectiveness of resilience-based programs for adolescents. Chandler et al. [7] reported significant improvements in resilience among adolescents in an intervention group compared with controls. Bluth and Eisenlohr-Moul [8] documented sustained improvements in resilience, self-compassion, and perceived stress from pre-intervention through follow-up. Associated benefits across other studies include enhanced coping and problem-solving skills [9], increased self-esteem, self-awareness, and social-emotional competence [10], reduced anxiety and depressive symptoms [11], and significant decreases in emotional distress [12].

The present study applies the Changing Minds, Changing Lives (CMCL) program developed by Chandler and Helling [13], a structured, strength-based resilience intervention grounded in Self-Determination Theory: SDT [14]. SDT posits that psychological well-being is supported by the fulfillment of three core needs: autonomy, competence, and relatedness. The CMCL program operationalizes these needs through the ABCs of resilience framework—Active coping, Building strength, and Cultivating connections [15]—delivered through a repeating sequence of centering practices, psychoeducation, structured reflective writing, and peer affirmation.

The CMCL program is well suited to Thai secondary school students for developmental, cultural, and educational reasons. Developmentally, adolescence is a critical period for identity formation and autonomy development, and the program’s strength-identification and reflective components directly support these tasks while addressing the SDT needs of autonomy, competence, and relatedness [14]. Culturally, its small-group sharing structure is congruent with Thai collectivist values, its centering practices (breathwork and mindfulness-based exercises) resonate with contemplative traditions familiar in Thai educational culture, and its strength-based, non-pathologizing framing offers a non-stigmatizing entry point to mental health content—a particularly important consideration for male adolescents, who show lower rates of help-seeking. Educationally, the six-session, 50 min format is compatible with standard Thai secondary school timetables and requires no clinical infrastructure.

Despite this growing international evidence base, three gaps remain. First, although the CMCL program has demonstrated positive outcomes among college athletes and young adults in the United States [7,15] and, in Thailand, among first-year nursing students in a pilot implementation, it has not previously been culturally adapted for and evaluated among Thai secondary school students. Second, male adolescents remain under-represented in school-based mental health intervention research, despite consistently lower rates of help-seeking and emotional disclosure; evidence on whether structured, strength-based group programs are acceptable and beneficial for this population is limited. Third, most prior evaluations have relied solely on quantitative outcome measures, leaving unexplained how and why change occurs from participants’ own perspectives. The present study addresses these gaps through a mixed-methods evaluation of the culturally adapted CMCL program among male secondary school students in Bangkok, Thailand. The study aimed to (1) quantitatively examine changes in resilience, perceived stress, and depressive symptoms from baseline to immediately post-intervention and one-month follow-up and (2) qualitatively explore participants’ experiences of the program and their perceptions of how it influenced them.

## 2. Materials and Methods

### 2.1. Study Design

A quasi-experimental, single-group, pre–post–follow-up design with a mixed-methods approach was employed. The quantitative component used repeated measures at three time points, while the qualitative component collected narrative data through in-class reflective writing and final group presentations. Specifically, a convergent mixed-methods design was used, in which quantitative and qualitative data were collected concurrently during the study period, analyzed separately, and integrated at the interpretation stage (see Section 2.10). Reporting follows relevant items of the TREND statement for transparent reporting of non-randomized intervention evaluations [16]. The study was conducted at a private, single-sex secondary school in Bangkok, Thailand, between November 2025 and March 2026. This study was conducted in accordance with the Declaration of Helsinki and approved by the Ramathibodi Ethics Committee, Faculty of Medicine Ramathibodi Hospital, Mahidol University.

### 2.2. Participants and Sampling

**Figure 1:**
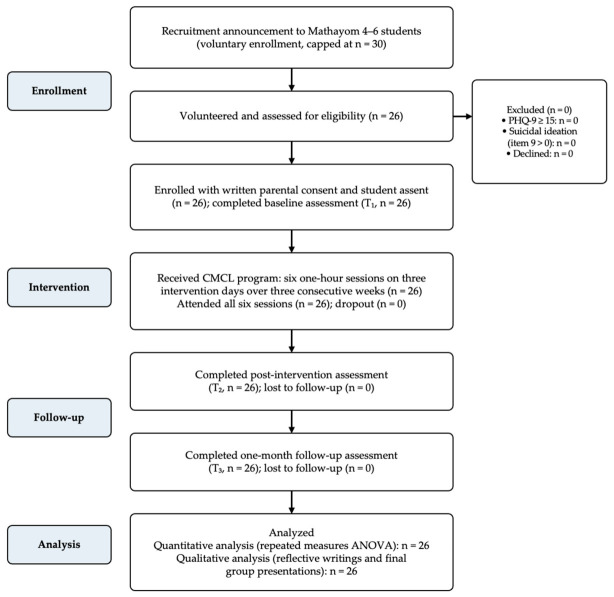
Participant flow diagram (TREND format).

The study samples comprised male secondary school students enrolled in Mathayom levels 4–6 (equivalent to grades 10–12), aged 15 years or older. Convenience sampling was used for recruitment. The program was announced to all Mathayom 4–6 students through the school’s student affairs channels, with participation open on a voluntary basis and enrollment capped at 30 places for pedagogical reasons (small-group format). Twenty-six students volunteered and were assessed for eligibility; none met exclusion criteria at baseline screening, and all 26 enrolled after written parental consent and student assent were obtained. All 26 participants attended all six sessions and completed all three assessment points (T_1_, T_2_, and T_3_), with no attrition. Participant flow is summarized in Figure 1.

Sample size was determined a priori using G*Power software (version 3.1) for a repeated measures ANOVA design with three time points, assuming a medium effect size (f = 0.25), an alpha level of 0.05, desired statistical power of 0.80, and a moderate correlation of 0.50 among repeated measures. The analysis yielded a required sample size of 26 participants, deemed sufficient to detect significant within-subject changes over time. This sample size calculation supports the detection of within-group change over time; it does not, in the absence of a control group, support causal conclusions about intervention effectiveness. For the qualitative strand, data adequacy was supported by the breadth and depth of the dataset: all 26 participants contributed reflective writings across six sessions in addition to final group presentations, yielding rich longitudinal narrative data. During analysis, no new codes emerged from the final portions of the dataset, indicating that the data were sufficient to support the themes identified.

Inclusion criteria required participants to be (1) currently enrolled in Mathayom 4–6 at a private secondary school in Bangkok; (2) aged 15 years or older at enrollment; (3) able to read and write Thai; (4) available to attend all six program sessions; and (5) demonstrating sufficient emotional stability to engage in a non-clinical group intervention. Participants were excluded if they (1) scored in the moderately severe to severe range on the PHQ-9 (≥15), indicating clinical risk; (2) exhibited active suicidal ideation as indicated by a positive response to PHQ-9 item 9 or as reported by a teacher, parent, or school counselor; (3) were currently experiencing severe psychological distress or psychiatric symptoms requiring clinical treatment; or (4) withdrew assent or had parental consent refused at any point. All participants completed the PHQ-9 as part of baseline screening prior to enrollment confirmation. Eligibility was verified individually before the first session; no prospective participant was excluded on clinical grounds.

### 2.3. Ethical Considerations

Written informed consent was obtained from parents or legal guardians, and written assent was obtained from all student participants prior to any data collection. The ethics-approved protocol and assent procedure covered adolescent participants aged 13–18 years. Participation was entirely voluntary, and withdrawal at any time was permitted without academic consequence. All data were de-identified using unique participant codes; a secure master list linking codes to identities was stored separately and accessible only to the principal investigator. Participants identified as experiencing significant psychological distress during the study were referred to appropriate school-based or external mental health services.

A pre-specified safety protocol was in place throughout the study. All PHQ-9 screenings, including item 9 (thoughts of self-harm or suicidal ideation), were reviewed individually by the principal investigator, a doctorally prepared psychiatric and mental health nursing faculty member. Any positive response to item 9, a total score of 15 or above, or concern raised by a teacher, parent, or school counselor would activate an individual risk assessment by the principal investigator, followed by referral to the school counseling service and, where clinically indicated, to psychiatric services at X Hospital, with parents or guardians informed in accordance with the ethics-approved procedures. All three facilitators were psychiatric and mental health nursing faculty members qualified to recognize and respond to psychological distress during sessions. No participant required activation of this protocol during the study. Confidentiality and its limits were explained to students and parents during the consent and assent process: reflective writings were read only by the research team and were not shared with teachers or parents; small-group sharing was voluntary, with participants free to choose what to disclose; and group ground rules of mutual respect and confidentiality were established in the first session. Students were informed that confidentiality would be broken only if there were concerns about safety.

### 2.4. Intervention: The CMCL Program

The CMCL resilience program used in this study was developed by Chandler and Helling [13] and delivered following the published facilitator manual, which specifies session objectives, scripts, psychoeducational content, reflective writing prompts, and homework assignments (Table 1). The intervention was adapted and delivered across six sessions over three consecutive weeks, on three intervention days (two sessions per day, separated by a 30 min break); each session was scheduled for one hour, comprising approximately 50 min of structured activity. A pre-class visit covered study information, consent and assent procedures, and the online baseline assessment; the final group presentations and the post-intervention assessment took place immediately after Session 6, and the follow-up assessment was completed online one month after program completion.

The program is structured around the ABCs of resilience framework [15] grounded in SDT [14], with each session following a consistent five-component sequence: (1) a centering practice (yoga or breathwork, 5 min); (2) a research presentation on a resilience-related topic (15 min); (3) structured reflective writing in response to a prompt (10 min); (4) small-group sharing with peer and instructor affirmative feedback (15 min); and (5) a closing 3A reflection—Affirmation, Appreciation, and Appraisal (5 min). Homework reinforcing positive practices and personal strengths identification was assigned after each session. A session-by-session structural overview and the Thai-language homework assignments used in this study are provided in Supplementary Material S1; the complete facilitator manual, including full session scripts and psychoeducational content, is proprietary to the program developers and is available from them [13].

The intervention was facilitated by a team of three psychiatric and mental health nursing faculty members. The principal investigator, who received formal training in the CMCL program from the program developers during her doctoral study and had previously implemented a pilot version among Thai first-year undergraduate nursing students as part of her doctoral dissertation, led the main sessions. The two co-investigators co-facilitated small-group activities, each overseeing a breakout group. All three team members had prior exposure to CMCL content through their teaching of a course on “Psychology for Personal Development” and received structured training from the principal investigator prior to implementation, totaling 8 h and covering program theory, session structure, facilitation of reflective writing, small-group sharing and affirmative feedback, and safety procedures. Fidelity was supported by adherence to the facilitator manual and the fixed five-component session structure, session checklists completed by the facilitation team after each session, and post-session team debriefings. All six sessions were delivered as planned, and no modifications to program content or structure occurred during implementation.

### 2.5. Translation and Cultural Adaptation

The CMCL materials were translated from the English-language facilitator manual [13] into Thai by the principal investigator, who is bilingual and was trained in program delivery by the program developers. Translation prioritized conceptual and cultural equivalence over literal rendering: psychoeducational examples and scenarios were adapted to the Thai secondary school context (e.g., academic and family situations familiar to Thai adolescents), reflective writing prompts were phrased in age-appropriate Thai, and centering practices were retained given their congruence with contemplative traditions familiar in Thai culture. The core components, session sequence, and theoretical structure of the program were preserved without modification. The adapted Thai-language program was reviewed by the research team, all of whom are psychiatric and mental health nursing faculty members experienced in adolescent mental health, and had previously been piloted by the principal investigator with Thai first-year undergraduate nursing students as part of her doctoral dissertation, which supported its acceptability, comprehensibility, and feasibility in a Thai educational setting prior to the present study. Formal back-translation was not performed; this is acknowledged as a limitation (Section 4.3).

### 2.6. Outcome Measures

All measures were completed online at three time points: baseline prior to the first session (T_1_), immediately following the final session (T_2_), and one month after program completion (T_3_).

Thai Connor–Davidson Resilience Scale (Thai CD-RISC) is a 25-item self-report instrument assessing resilience, originally developed by Connor and Davidson [17]. The CD-RISC uses a 5-point Likert scale (0–4), with total scores ranging from 0 to 100. Higher scores reflect greater resilience. The original CD-RISC demonstrated good internal consistency (α = 0.89) and a five-factor structure across multiple clinical and community samples [17]. The Thai-translated CD-RISC 25-item has been applied in Thai youth samples and demonstrated excellent internal consistency (α = 0.96, *n* = 30) by Inoura et al. [18] and acceptable psychometric properties with a three-factor structure in the Thai context [19].

Thai Perceived Stress Scale (T-PSS-10) is a 10-item self-report questionnaire assessing the degree to which life situations are perceived as stressful over the past month. This measure was developed by Cohen et al. [20] as a 14-item global measure of perceived stress, subsequently shortened to 10 items (PSS-10) through factor analysis. Items are rated on a 5-point Likert scale (0–4); total scores range from 0 to 40, with higher scores indicating greater perceived stress. The Thai version (T-PSS-10) was translated and validated by Wongpakaran and Wongpakaran [21], demonstrating excellent internal consistency (α = 0.85) in a Thai adult sample.

Thai Patient Health Questionnaire-9 (PHQ-9) is a 9-item self-report instrument developed by Kroenke et al. [22] to screen for depressive symptoms over the previous two weeks. Each item is rated on a 4-point Likert scale (0–3); total scores range from 0 to 27, with higher scores indicating greater symptom severity. The Thai version was translated and validated by Lotrakul et al. [23], demonstrating internal consistency (α = 0.79) in adult primary care patients. In the present study, the Thai PHQ-9 (adult version) [23] was administered. Although an adolescent adaptation (PHQ-A) has been validated in Thai adolescent psychiatric patients [24], demonstrating that PHQ-based depression screening performs reliably in Thai adolescents (α = 0.92), the Thai PHQ-9 was selected because it is brief, is well validated in the Thai language, and demonstrated acceptable internal consistency in the present sample (α = 0.788). The use of the adult version with an adolescent sample is acknowledged as a limitation (Section 4.3).

### 2.7. Reliability of Instruments in This Sample

Internal consistency of all three instruments was assessed in the present sample prior to the main analyses using Cronbach’s alpha calculated from the baseline (T_1_) administration, prior to the main analyses. The Thai CD-RISC demonstrated excellent internal consistency (α = 0.921), the T-PSS-10 demonstrated acceptable internal consistency (α = 0.772), and the PHQ-9 demonstrated acceptable internal consistency (α = 0.788). These values are consistent with those reported in the original Thai validation studies [18,19,21,23,24], confirming that all three instruments performed reliably within this sample.

### 2.8. Quantitative Statistical Analysis

All analyses were performed using IBM SPSS Statistics version 31.0 [25]. All 26 participants completed all three assessments, with no missing assessment points. Prior to the main analyses, minimal item-level missing data were handled using participant-level mean imputation, whereby missing values within a scale were replaced with the participant’s mean across the completed items on that scale. Descriptive statistics (mean, standard deviation, frequency, and percentage) were calculated to summarize participant characteristics and baseline scores. Repeated measures ANOVA was conducted for each outcome variable (Thai CD-RISC, T-PSS-10, PHQ-9) across the three time points. Prior to analysis, data were screened for normality using the Shapiro–Wilk test and for sphericity using Mauchly’s test; where sphericity was violated, the Greenhouse–Geisser correction was applied. Statistically significant time effects were followed by Bonferroni-corrected post hoc pairwise comparisons, with Bonferroni-adjusted 95% confidence intervals reported for all pairwise mean differences. Effect sizes are reported as partial eta squared (η^2^). The significance level was set at α = 0.05. Because the dataset was complete and balanced across the three time points, repeated measures ANOVA was retained as the primary analysis; under these conditions, a linear mixed model yields equivalent fixed effects estimates and offers no additional advantage in handling missing data.

### 2.9. Qualitative Analysis

Qualitative data from in-class reflective writing exercises and final group presentations were analyzed using conventional content analysis [26], attending to both manifest and latent content. All 26 participants completed a brief written reflection (approximately 100–150 words) at the end of each of the six sessions, yielding 156 reflective texts in total, with no missing reflections; the analyzed corpus comprised these 156 texts together with the 26 final presentations (one per participant, delivered to the whole group in Session 6). All data were originally produced in Thai, and coding was conducted in Thai; selected quotations were translated into English by the bilingual principal investigator and verified for accuracy of meaning by a second bilingual member of the research team. An inductive approach was employed, beginning with line-by-line coding to capture key expressions and meanings. Similar codes were iteratively grouped into categories and refined into overarching themes through comparative abstraction. Two researchers independently coded the data; discrepancies were resolved through team discussion until full consensus was reached. Because coding reliability was established through this consensus-based (dialogic) approach rather than through independent duplicate rating, a quantitative intercoder agreement coefficient was not calculated, consistent with methodological guidance for conventional content analysis [27]. Trustworthiness was enhanced through multiple-coder cross-checking, peer debriefing, and member checking, in which the preliminary themes, together with illustrative extracts, were presented to three participants, who confirmed that the themes accurately reflected their experiences; no modifications were required. The full coding framework (codes, categories, and themes, with example data extracts) is provided in Supplementary Material S2.

Reflexivity: the facilitators of the intervention were also members of the research team, a dual role that may have influenced participants’ written and spoken accounts through social desirability. To mitigate this, reflective writing was framed as a personal learning exercise rather than a program evaluation, sharing in groups was voluntary, students were assured that their reflections would not affect any school evaluation, and the analysis deliberately attended to accounts that diverged from expected patterns.

### 2.10. Quantitative and Qualitative Data Integration

A convergent mixed-methods approach was used to integrate quantitative and qualitative findings during interpretation [28]. Quantitative data addressed whether and to what degree changes occurred in stress, depression, and resilience; qualitative data provided insight into how and why these changes occurred. The two strands were collected concurrently during the study period but analyzed separately and independently. Integration occurred at the interpretation stage, after both analyses were complete, using a triangulation protocol in which quantitative outcomes and qualitative themes were compared for convergence, complementarity, and divergence. The results of this integration are presented in a joint display, which maps each quantitative outcome to its converging qualitative theme(s) and the resulting meta-inference.

## 3. Results

### 3.1. Participant Characteristics

All 26 enrolled participants attended all six program sessions and completed all three assessment points; participant flow is shown in Figure 1. The mean age was 16.08 years (SD = 0.74, range 15–18). Regarding gender identity, 24 participants (92.3%) identified as male, and 2 (7.7%) did not specify gender. All participants were students at a single-sex boys’ school; gender identity was additionally assessed by self-report, with “prefer not to say” offered as a standard response option, which two participants selected. In terms of grade level, 12 participants (46.2%) were enrolled in Mathayom 4 (Grade 10) and 14 (53.8%) in Mathayom 5 (Grade 11). Although Mathayom 6 students were eligible, none enrolled, most plausibly because the study period coincided with their preparation for university entrance examinations. The majority of participants (*n* = 20, 80.0%) reported living with both parents, 3 (12.0%) lived with one parent, and 2 (8.0%) lived with relatives (one participant had missing data for this item). When experiencing stress or distress, 12 participants (46.2%) reported consulting both family and individuals outside the family, 5 (19.2%) consulted family members only, 5 (19.2%) kept their feelings to themselves, and 4 (15.4%) consulted only individuals outside the family. The mean daily social media usage was 5.92 h (SD = 2.87, range 1–10 h), and the mean cumulative GPA was 3.50 (SD = 0.29, range 3.00–3.96).

Baseline PHQ-9 screening confirmed that no participant scored 15 or above, and no participant endorsed suicidal ideation (PHQ-9 item 9 = 0 for all participants), confirming that all enrolled participants met the eligibility criteria for participation in a non-clinical intervention. Full participant characteristics are presented in Table 2.

### 3.2. Quantitative Outcomes

**Figure 2:**
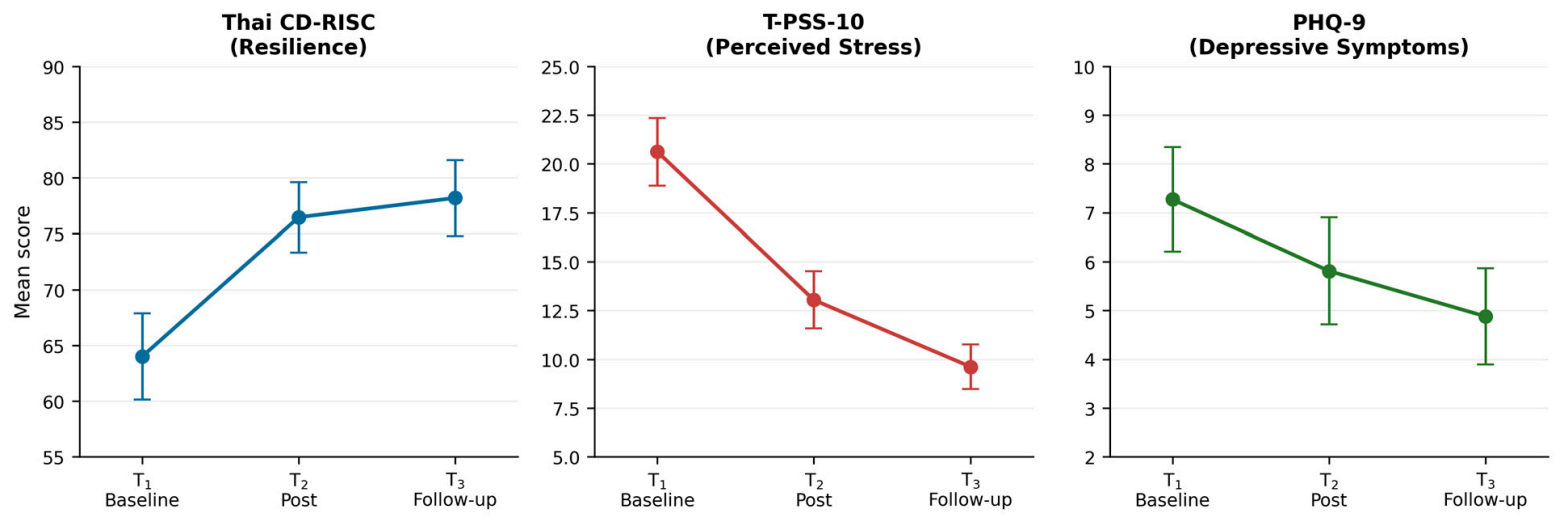
Mean scores (±95% confidence intervals) for resilience (Thai CD-RISC), perceived stress (T-PSS-10), and depressive symptoms (PHQ-9) at baseline (T_1_), post-intervention (T_2_), and one-month follow-up (T_3_); *N* = 26.

Descriptive statistics for all three outcome measures across the three time points are presented in Table 3, and outcome trajectories are shown in Figure 2. Prior to the repeated measures ANOVA, Mauchly’s test of sphericity was conducted for each outcome. Sphericity was met for resilience (Thai CD-RISC; W = 0.899, χ^2^(2) = 2.54, *p* = 0.280) and for depressive symptoms (PHQ-9; W = 0.810, χ^2^(2) = 5.05, *p* = 0.080); therefore, sphericity-assumed values are reported for both outcomes. Sphericity was violated for perceived stress (T-PSS-10; W = 0.723, χ^2^(2) = 7.77, *p* = 0.021); therefore, the Greenhouse–Geisser correction was applied for that outcome.

Resilience scores increased significantly over time (*F*_(2,50)_ = 75.63, *p* < 0.001, η^2^ = 0.752, large effect). Bonferroni-corrected post hoc comparisons revealed in Table 4 that resilience was significantly higher at T_2_ (*M* = 76.46, SD = 7.75) compared with T_1_ (*M* = 64.00, SD = 9.58; mean difference = 12.46, *p* < 0.001), and significantly higher at T_3_ (*M* = 78.19, SD = 8.43) compared with T_1_ (mean difference = 14.19, *p* < 0.001). The difference between T_2_ and T_3_ was not statistically significant (mean difference = 1.73, *p* = 0.327), indicating that resilience gains were achieved by post-intervention and maintained at one-month follow-up.

Perceived stress scores decreased significantly over time (*F*_(1.567,39.17)_ = 105.03, *p* < 0.001, η^2^ = 0.808, large effect). Post hoc comparisons showed in Table 4 that perceived stress was significantly lower at T_2_ (*M* = 13.04, SD = 3.63) compared with T_1_ (*M* = 20.62, SD = 4.30; mean difference = 7.58, *p* < 0.001), and significantly lower still at T_3_ (*M* = 9.62, SD = 2.80) compared with both T_1_ (mean difference = 11.00, *p* < 0.001) and T_2_ (mean difference = 3.42, *p* < 0.001), indicating a continued and progressive reduction in perceived stress through the follow-up period.

Depressive symptoms also decreased significantly over time (*F*_(2,50)_ = 14.74, *p* < 0.001, η^2^ = 0.371, moderate-to-large effect). Post hoc comparisons indicated in Table 4 that PHQ-9 scores were significantly lower at T_2_ (*M* = 5.81, SD = 2.70) compared with T_1_ (*M* = 7.27, SD = 2.65; mean difference = 1.46, *p* = 0.001), and significantly lower at T_3_ (*M* = 4.88, SD = 2.44) compared with T_1_ (mean difference = 2.38, *p* < 0.001). The difference between T_2_ and T_3_ was not statistically significant (mean difference = 0.92, *p* = 0.129), indicating that the reduction in depressive symptoms was substantially achieved by post-intervention and maintained at one-month follow-up.

### 3.3. Qualitative Findings

Conventional content analysis of written reflective exercises and final group presentations from all 26 participants yielded three overarching themes: Self-Understanding, Stress Management, and Understanding Others. These themes and their representative codes are presented in Table 5. Themes were derived from patterns of meaning across the dataset rather than from frequency of occurrence; participant identifiers are reported to indicate the breadth of the supporting data. Unless otherwise indicated, quotations are drawn from participants’ reflective writings.

Theme 1: Self-Understanding

Self-Understanding was the dominant theme across the dataset, emerging as the primary theme for the majority of participants and present in all 26 participants when both primary and secondary data are considered. This theme encompassed recognizing strengths and weaknesses, recognizing one’s own emotions and feelings, gaining self-compassion, developing new self-perceptions, and cultivating a sense of self-worth. Participants described how they recognized their own feelings, emotions, strengths, and weaknesses, subsequently developing self-compassion and self-worth throughout the program. Consequently, they gained enhanced self-understanding through an expanded view of themselves. Most participants reported they understand themselves rather than explore strength or weakness and recognize their own feelings (P01, P03, P04, P08, P10, P11, P16, P17, P18, P19, P21, P24, P26). Others represented self-compassion: ‘I learn to love and be kind to myself, let something terrible away’ (P10, P20). One described at final reflection: ‘I learn to thank myself and feel worthy of me’ (P22). One participant summarized this recognition succinctly: ‘No one understands us better than we understand ourselves’ (P01). Another articulated a future-oriented expression of this self-understanding: ‘In the future if I feel discouraged, I will try to look at myself more carefully’ (P26). These findings represented comprehensive self-understanding through expanded self-perception, integrating emotional awareness, personal acceptance, and enhanced self-worth. This holistic self-understanding represents a fundamental therapeutic outcome that underpins improved psychological well-being and adaptive functioning.

Theme 2: Stress Management

Stress management is defined as alleviating negative emotions through modified coping strategies, positive thinking, assertiveness development, problem-solving approaches, and venting negative feelings with friends. Participants acquired stress management techniques through peer observation and discussion, adapting strategies to their individual circumstances: “I listen to my friends’ story then thinking about the ways dealing with stress and adapt to mine” (P2, P3, P7, P10, P13, P15, P16, P17, P21, P25). Several participants (P19, P23, P24, P25) reported enhanced assertiveness skills through program activities, enabling better boundary-setting and self-advocacy. Participants demonstrated adoption of positive thinking patterns: “I have positive thinking when stressful” (P7, P12, P17, P26). The program fostered metacognitive development, with one participant stating: “I have insight to my problem and know how to deal with it” (P14). Participants experienced benefits from authentic self-disclosure: “I have a chance to express my stress sincerely to myself and feel better afterwards” (P21). These findings indicate that comprehensive stress management programs can enhance adaptive coping capacities through peer learning, skill development, cognitive restructuring, and emotional processing opportunities.

Theme 3: Understanding Others

Understanding others emerged as a theme within the transcripts, though with less frequency than self-understanding. Nevertheless, this theme represents significant growth in participants’ interpersonal awareness and empathetic capacity. The cultivation of careful listening skills enabled participants to gain insight into others’ motivations and behavioral patterns. As articulated by participants: “I carefully listen to my friends’ stories then I understand why they do things this way. This makes me more open minded towards others” (P3, P4, P9, P19). This relational growth was also expressed as a felt sense of connection during the final group presentations: “We are not alone; there is always someone with us” (P04, final presentation). This finding indicates that structured opportunities for narrative sharing and active listening can foster meaningful interpersonal understanding. The process of deeply engaging with others’ experiences resulted in increased cognitive flexibility and reduced judgmental attitudes. Participants reported greater acceptance and understanding of diverse perspectives, suggesting that empathetic listening serves as a mechanism for developing tolerance and open-mindedness.

### 3.4. Mixed-Methods Integration

Integration of quantitative and qualitative findings through a convergent mixed-methods triangulation approach yielded a coherent, mutually reinforcing, and theoretically grounded account of how the CMCL program may have produced the observed changes. Quantitative data established that significant within-group change occurred across all three outcomes; qualitative data illuminated the subjective processes through which participants experienced and made sense of that change. Together, the two data strands produced a richer and more actionable evidence base than either approach alone could generate [28]. The integration is summarized in a joint display (Table 6).

The most direct quantitative–qualitative convergence is observed between the perceived stress findings and Theme 2 (Stress Management). Perceived stress showed the largest effect (η^2^ = 0.808) and the unique pattern of continued decline through T_3_, suggesting that change did not end with the program but extended into everyday life. This converges with participants’ accounts of adopting and adapting coping strategies beyond the classroom: “I listen to my friends’ story then thinking about the ways of dealing with stress and adapt to mine” (P02, P03, P10); “I have positive thinking when stressful” (P07, P12, P17, P26). One interpretation, suggested by the qualitative data although not directly tested, is that participants continued to apply peer-learned reappraisal skills after the program ended—an active, ongoing cognitive process embedded socially through the peer-sharing structure—which would be consistent with the continued decline in T-PSS-10 scores through follow-up.

The Thai CD-RISC gains (η^2^ = 0.752) converge most directly with Theme 1 (Self-Understanding). The quantitative increase in resilience across T_1_ to T_3_ is mirrored in the qualitative shift from limited self-knowledge to expanded self-perception that permeated participants’ reflective writing. Participants described recognizing their own emotions and strengths in ways they had not before, developing self-compassion—“I learn to love and be kind to myself, let something terrible away” (P10, P20)—and developing future-oriented coping intentions: “In the future if I feel discouraged, I will try to look at myself more carefully” (P26). These qualitative accounts describe the internal architecture of resilience: the capacity to recognize one’s own resources, extend self-compassion, and mobilize those resources prospectively. The Thai CD-RISC measures precisely this capacity. The qualitative data thus suggest an interpretive account, grounded in participants’ own perspectives, of how resilience gains may have arisen: through a shift in how participants related to themselves.

The PHQ-9 reductions (η^2^ = 0.371) find qualitative support distributed across all three themes, reflecting the multidimensional psychosocial nature of subclinical depression in adolescents. Theme 1 contributes through self-compassion and increased self-worth, which may counter the negative self-evaluation central to depressive cognition. Theme 2 contributes through assertiveness development—several participants (P19, P23, P24, P25) reported enhanced capacity for self-advocacy and boundary-setting, competencies that may reduce the passive helplessness associated with depression. Theme 3 contributes most distinctively: the reduction in perceived isolation captured in “We are not alone; there is always someone with us” (P04), and the development of empathetic listening skills enabling participants to feel genuinely understood by peers, may help address the social disconnection that underlies much adolescent depressive affect. While the quantitative PHQ-9 scores show the magnitude of change, the qualitative data suggest a possible relational pathway—the experience of being heard and of genuinely hearing others—although this interpretation was not directly tested in the present design.

Beyond these three convergences, the qualitative data revealed impacts that the quantitative instruments were not designed to capture. Transfer of learning to family contexts—P23 reporting greater willingness to communicate with parents, P03 applying program skills at home—represents a broadening of the program’s effects beyond the school setting. P26’s explicit articulation of a future coping intention represents the development of what might be termed a ‘resilience identity’: an internalized self-conception as someone capable of coping. These qualitative findings point to program effects that may be broader, more durable, and more personally embedded than any single outcome score can represent. Taken together, quantitative and qualitative strands do not merely confirm each other; they complete each other, producing an integrated understanding of both the observed changes and participants’ perceptions of how those changes occurred.

## 4. Discussion

### 4.1. Changes Associated with the CMCL Strength-Based Resilience Program

This study evaluated changes in resilience, perceived stress, and depressive symptoms associated with participation in the CMCL strength-based resilience program among secondary school students in Bangkok, Thailand. Findings showed large and statistically significant within-group improvements across all three measured outcomes—resilience, perceived stress, and depressive symptoms—with changes maintained at one-month follow-up. Qualitative analysis yielded three overarching themes—Self-Understanding, Stress Management, and Understanding Others—that together provide an interpretive account of the processes through which the program may have contributed to these changes. The convergence of quantitative and qualitative evidence provides preliminary support for the potential of structured resilience programs in Thai secondary schools. Because the study lacked a control group, however, the observed changes cannot be attributed solely to the program; maturation, repeated testing, expectancy effects, and contextual factors may have contributed, and the findings should therefore be interpreted as preliminary.

The large effect size for resilience (η^2^ = 0.752) suggests a substantial within-group improvement in participants’ capacity to cope with adversity, consistent with the program’s theoretical grounding in SDT and the ABCs of resilience framework. The mean resilience score increased by more than 14 points across the study period, and gains were not reversed at follow-up (*p* = 0.327 for T_2_ vs. T_3_), which may indicate that program-acquired skills and mindsets continued to consolidate after formal completion. This durability aligns with prior evaluations of strength-based resilience interventions [7,8] and with the broader evidence base on school-based resilience programs demonstrating sustained effects [10]. These quantitative gains are directly mirrored in Theme 1 (Self-Understanding), the most prevalent theme across the dataset. Participants described enhanced recognition of their own emotions, strengths, and limitations, alongside the development of self-compassion and self-worth. The codes of self-reflection, strength identification, and increased self-worth map precisely onto the internal resources that resilience theory identifies as foundational to adaptive coping [14,15]. Notably, participants reported gains in self-compassion—“I learn to love and be kind to myself, let something terrible away” (P10, P20)—a finding consistent with the evidence that self-compassion is a key mediator of improved psychological well-being and reduced depression in adolescents [8]. The sense of expanded self-perception articulated by P22—“I learn to thank myself and feel worthy of me”—reflects the deeper psychological shift from deficit awareness to personal affirmation that the CMCL program is specifically designed to cultivate.

The very large effect size for perceived stress (η^2^ = 0.808) is the most striking quantitative finding of this study. Uniquely, perceived stress demonstrated a progressive pattern of decline across all three time points, with a further significant decrease between T_2_ and T_3_ (*p* < 0.001), indicating that stress reduction continued to develop beyond the program period itself. One plausible interpretation, supported by the qualitative data although not directly tested, is the sustained application of cognitive reappraisal strategies in everyday contexts—identified across the literature as a core active ingredient in resilience and stress management interventions, enabling individuals to reinterpret adverse experiences in a more adaptive manner and thereby enhance resilience [5]. Theme 2 (Stress Management) converges with this interpretation. Participants reported learning to modify their coping strategies through peer observation: “I listen to my friends’ story then thinking about the ways of dealing with stress and adapt to mine” (P02, P03, P10). Several described the adoption of positive thinking patterns—“I have positive thinking when stressful” (P07, P12, P17, P26)—and the development of metacognitive insight: “I have insight to my problem and know how to deal with it” (P14). One participant described emotional relief that epitomises the cathartic and regulatory dimension of the program: “I have a chance to express my stress sincerely to myself and feel better afterwards” (P21). These qualitative accounts are consistent with the study by Llistosella et al. [29], whose cluster-RCT of a comparable school-based resilience intervention similarly identified emotional regulation and cognitive reappraisal as the primary theoretical targets for sustained stress reduction in adolescents. The progressive stress reduction beyond T_2_ is consistent with participants having continued to apply coping frameworks after program completion, although this interpretation could not be tested directly in the present design. It should also be noted that the T-PSS-10 assesses perceived stress over the preceding month, whereas the intervention lasted three weeks; the T_2_ recall window therefore partially overlapped the pre-intervention period, and may conservatively underestimate change during the program. The further decline at T_3_, whose recall window fell entirely after program completion, is not attributable to this overlap.

Depressive symptoms decreased significantly (η^2^ = 0.371), with mean PHQ-9 scores at follow-up (*M* = 4.88) in the minimal symptom range, underscoring the preventive potential of the program for non-clinical adolescent populations. The moderate-to-large effect size is consistent with a preventive rather than therapeutic intervention, targeting subclinical vulnerability rather than clinical disorder. The clinical relevance of these changes should be interpreted in context: the baseline means (*M* = 7.27) fell within the mild symptom range (5–9), and the mean reduction of 2.38 points by follow-up, while statistically significant, is modest in absolute terms and below thresholds typically considered clinically meaningful change in treatment samples. This pattern is expected for a preventive intervention delivered to a non-clinical population, in which floor effects constrain the magnitude of possible reduction; by follow-up, the group mean had moved into the minimal range (<5), indicating movement toward minimal symptomatology rather than clinical remission of disorder. This reduction is supported and enriched across all three qualitative themes. Theme 1 (Self-Understanding) contributes through the development of self-compassion and self-worth, both established buffers against depressive affect in adolescents [8]. Theme 2 (Stress Management) contributes through assertiveness development and behavioral activation: several participants (P19, P23, P24, P25) reported enhanced assertiveness skills enabling better boundary-setting and self-advocacy, protective competencies that may reduce the social helplessness associated with depression. Theme 3 (Understanding Others) contributes through the reduction in social isolation and the cultivation of peer connection—established risk factors for adolescent depression [4]. The program’s group-based small-sharing structure was particularly impactful here: participants’ descriptions of careful listening enabling greater understanding of others’ motivations—“I carefully listen to my friends’ stories then I understand why they do things this way. This makes me more open-minded towards others” (P03, P04, P09, P19)—reflect the cultivation of empathetic listening as a relational skill that may reduce perceived isolation and strengthen social support, both protective against depression.

The qualitative data also revealed possible processes that quantitative outcome measures alone could not capture. Participants consistently reported transfer of learning beyond the program context: applying stress management strategies at home, developing greater willingness to communicate with parents (P23), and articulating explicit future coping intentions—“In the future if I feel discouraged, I will try to look at myself more carefully” (P26). This generalization of skills across settings is a defining feature of educationally effective interventions and strengthens the argument for curriculum integration rather than one-off delivery. One participant directly credited the facilitator’s accessible style and engaging materials as enabling his engagement with the group sharing process (P06)—an observation that points to implementation quality as a critical moderator of program effectiveness, consistent with the finding that interventions delivered by mental health professionals or researchers produce significantly larger effects than those delivered by teachers [30]. An incidental observation during analysis further enriches interpretation: two participants (P13, P22) described limited prior engagement with emotion regulation or stress management, yet both participated meaningfully in the program. Although negative case analysis was not a pre-specified analytic step, this observation suggests that the CMCL program may be accessible to adolescents with a low baseline in coping readiness—an encouraging signal for population-level mental health programming, where the most vulnerable students are often those least likely to engage. Finally, because the facilitators were also members of the research team, participants’ accounts may partly reflect social desirability; this dual role is acknowledged as a limitation and was mitigated as described in Section 2.9.

### 4.2. Implications for School Policy and Practice

These findings have potential implications for school mental health policy in Thailand. The CMCL program’s format—six 50 min sessions over three weeks—is compatible with secondary school timetables, requires no clinical infrastructure, and could realistically be implemented by school nurses and school counselors, or by teachers who receive structured training and ongoing supervision from mental health professionals, given evidence that facilitator background moderates program effects [30]. Its group-based structure aligns with Thai collectivist values, its reflective writing component provides a non-stigmatizing emotional outlet, and its mindfulness-based centering practices draw on traditions familiar in Thai educational culture. Thailand’s Ministry of Education and Ministry of Public Health have both identified adolescent mental health as a national priority and called for school-level preventive programs [3]; the CMCL program represents a culturally consonant candidate whose inclusion in national school health curricula should follow confirmation of its effectiveness in controlled studies with larger and more diverse samples, longer follow-up, and systematic assessment of implementation fidelity. The mixed-methods evaluation framework employed in this study provides a replicable model for future implementations, enabling assessment not only of whether programs work but also of how and for whom. Furthermore, future iterations of the CMCL program should incorporate periodic booster sessions to reinforce resilience skills, sustain reductions in perceived stress, and prevent relapse of depressive symptoms over time, which may strengthen the long-term impact of school-based mental health programming.

### 4.3. Limitations

Several limitations should be considered. First, the quasi-experimental single-group design without a control group limits causal inference; the observed changes may partly reflect maturation, repeated testing, expectancy effects, or contextual factors such as the school calendar, and effect sizes estimated from small, single-group studies are susceptible to inflation and should be interpreted as indicative of within-group change over time rather than as unbiased estimates of intervention effectiveness. Second, the sample was drawn exclusively from a private single-sex male school in Bangkok, resulting in a homogeneous sample that restricts generalizability to female students, students from public schools, and those from lower socioeconomic or rural backgrounds. Third, several measurement limitations apply: the T-PSS-10 assesses stress over the preceding month, so the post-intervention recall window partially overlapped the pre-intervention period; the adult Thai PHQ-9, rather than the adolescent-specific PHQ-A, was administered; and formal back-translation of the culturally adapted program materials was not performed. Fourth, the facilitators were also members of the research team, and participants’ qualitative accounts may partly reflect social desirability. Lastly, the one-month follow-up period is insufficient to assess the durability of intervention effects across an academic year or beyond; longer-term assessment is needed to determine whether gains in resilience and reductions in stress and depressive symptoms are maintained.

Future research should employ randomized controlled trial designs with active control groups, larger and more diverse samples including both male and female students, and extended follow-up periods of at least six months to generate stronger causal evidence. Additionally, replication across varied school types is needed to examine the generalizability within diverse Thai secondary school contexts. Furthermore, future iterations of the program should incorporate periodic booster sessions to enhance the long-term effectiveness of strength-based resilience programming by maintaining resilience gains and sustaining reductions in perceived stress and depressive symptoms.

## 5. Conclusions

In this single-group pre–post–follow-up mixed-methods study, participation in the culturally adapted CMCL strength-based resilience program, grounded in SDT and the ABCs of resilience framework, was associated with increased resilience and reduced perceived stress and depressive symptoms among male students at a Thai private secondary school, with changes maintained at one-month follow-up. By integrating centering practices, psychoeducation, structured reflective writing, and peer acknowledgment, the program addresses core psychological needs of autonomy, competence, and relatedness. The qualitative findings—self-understanding, stress management, and understanding others—offer an interpretive account, grounded in participants’ own perspectives, of how these changes may have occurred. These preliminary findings support the feasibility, acceptability, and promise of the program in the Thai secondary school context, but do not establish its effectiveness. Controlled studies with larger and more diverse samples, longer follow-up, and systematic assessment of implementation fidelity are needed before broader implementation can be recommended.

## Figures and Tables

**Table 1:** CMCL Program Session Structure.

Sessions	Centering Practice	Research Topic	Writing Prompt	Refl.	Homework
1	Physicalize the Resilience Response	Introduction & Pre-Assessment	Letter to my future self/ I am from…	AAA	PP + StrengthQuest
2	Cloud Arms	Resilience	A time I was resilient	AAA	PP: Gratitude + Strength
3	Body Scan	Neurobiology of Stress	A time, a friend or I felt stressed	AAA	PP: Recover + Strength
4	Tree Pose	Stress, Health, Adversity & Resilience	Objects	AAA	PP: Focus + Strength
5	Four Element	Automatic Thinking	A recent experience with automatic thinking	AAA	PP: Joy + Strength
6	Loving Kindness	Self-Compassion Final Presentation	Goodness Cloud	AAA	Final Resilience Reflection

Note. PP = Positive Practice; Refl. = Reflection, AAA = Affirmation, Appreciation, Appraisal. Sessions were delivered twice weekly over three weeks. CMCL = Changing Minds, Changing Lives.

**Table 2:** Participant Characteristics (*N* = 26).

Characteristic	*n*	%	Mean ± SD (Range)
Age (years)			
Mean ± SD (Range)			16.08 ± 0.74 (15–18)
Gender identity ^a^			
Male	24	92.3	
Did not specify	2	7.7	
Grade level			
Mathayom 4 (Grade 10)	12	46.2	
Mathayom 5 (Grade 11)	14	53.8	
Current living arrangement (*n* = 25) ^b^			
Both parents	20	80.0	
One parent (father or mother)	3	12.0	
Relatives	2	8.0	
Help-seeking when stressed			
Family and others (both)	12	46.2	
Family only	5	19.2	
Keep to self	5	19.2	
Outside family only	4	15.4	
Daily social media usage (hours)			
Mean ± SD (Range)			5.92 ± 2.87 (1–10)
Cumulative GPA (*n* = 25) ^b^			
Mean ± SD (Range)			3.50 ± 0.29 (3.00–3.96)

Note. ^a^ Gender identity was self-reported, with “prefer not to say” offered as a standard response option; all participants were enrolled at a single-sex boys’ school. ^b^ One participant had missing data for this item.

**Table 3:** Means, Standard Deviations, and Repeated Measures ANOVA Results for Outcome Variables Across Three Time Points (*N* = 26).

Measure	T_1_ Baseline *M* (SD)	T_2_ Post *M* (SD)	T_3_ Follow-Up *M* (SD)	*F*	df	*p*	η^2^
Thai CD-RISC (Resilience)	64.00 (9.58)	76.46 (7.75)	78.19 (8.43)	75.63	2, 50	<0.001	0.752
T-PSS-10 (Perceived Stress)	20.62 (4.30)	13.04 (3.63)	9.62 (2.80)	105.03	1.567, 39.17 ^a^	<0.001	0.808
PHQ-9 (Depressive Symptoms)	7.27 (2.65)	5.81 (2.70)	4.88 (2.44)	14.74	2, 50	<0.001	0.371

Note. *M* = mean; SD = standard deviation; η^2^ = partial eta squared. ^a^ Greenhouse–Geisser correction applied due to violation of sphericity (Mauchly’s W = 0.723, *p* = 0.021).

**Table 4:** Bonferroni-Corrected Post Hoc Pairwise Comparisons Between Time Points.

Measure	Comparison	Mean Diff.	SE	*p* ^a^	95% CI ^b^
Thai CD-RISC	T1 → T2	+12.46	1.37	<0.001	[8.95, 15.98]
	T1 → T3	+14.19	1.34	<0.001	[10.75, 17.63]
	T2 → T3	+1.73	1.04	0.327	[−0.94, 4.40]
T-PSS-10	T1 → T2	−7.58	0.76	<0.001	[−9.52, −5.64]
	T1 → T3	−11.00	0.95	<0.001	[−13.42, −8.58]
	T2 → T3	−3.42	0.59	<0.001	[−4.93, −1.92]
PHQ-9	T1 → T2	−1.46	0.36	0.001	[−2.38, −0.55]
	T1 → T3	−2.38	0.52	<0.001	[−3.73, −1.04]
	T2 → T3	−0.92	0.43	0.129	[−2.03, 0.19]

Note. ^a^ Bonferroni-corrected *p*-values. SE = standard error. ^b^ Bonferroni-adjusted 95% confidence intervals for the pairwise mean differences.

**Table 5:** Themes and Frequencies from Conventional Content Analysis of Written Reflections (*N* = 26).

#	Theme	Representative Codes
1	Self-Understanding	Recognize strength and weakness, Recognizing own emotions and feelings, Gaining self-compassion, Opening a new self-perception, Having sense of self-worth
2	Stress Management	Learning and modified various ways of coping methods, positive thinking, Assertiveness, Problem-Solving approach, Venting negative feelings
3	Understanding Others	Empathetic listening to others, Gaining understand others’ view

**Table 6:** Joint Display of Mixed-Methods Integration: Convergence of Quantitative Outcomes and Qualitative Themes.

Quantitative Finding	Converging Qualitative Theme (Illustrative Codes)	Meta-Inference (Interpretation)
Resilience (Thai CD-RISC) increased from T1 to T3 (η^2^ = 0.752); gains achieved by T2 and maintained at follow-up	Theme 1: Self-Understanding (recognizing strengths and emotions; self-compassion; self-worth; new self-perception)	Resilience gains converge with participants’ accounts of expanded self-perception; self-understanding may constitute the internal foundation of resilience (interpretation, not directly tested)
Perceived stress (T-PSS-10) decreased progressively across all time points, including T2 to T3 (η^2^ = 0.808)	Theme 2: Stress Management (adapted coping strategies; positive thinking; assertiveness; venting negative feelings)	Continued post-program decline converges with accounts of ongoing application of peer-learned coping and reappraisal strategies in everyday life
Depressive symptoms (PHQ-9) decreased from T1 to T3 (η^2^ = 0.371); follow-up mean in minimal range	Themes 1–3 (self-worth; assertiveness and self-advocacy; connectedness and being heard)	Symptom reduction converges with increased self-worth, self-advocacy, and reduced perceived isolation; the relational experience of being heard may buffer depressive affect
Not captured by quantitative instruments	Transfer of skills to family contexts (P03, P23); future coping intentions (P26)	The qualitative strand expands the quantitative findings, pointing to broader and more personally embedded change beyond measured outcomes

## Data Availability

The data presented in this study are available on request from the corresponding author. The data are not publicly available due to privacy and ethical restrictions related to the participation of minors.

## Supplementary Materials

The following supporting information can be downloaded at: https://www.mdpi.com/article/10.3390/healthcare14152337/s1. Supplementary Material S1: CMCL Program: Delivery Schedule, Session Structure, and Thai-Language Class Worksheets and Homework (the complete facilitator manual is proprietary to the program developers and available from them [13]); S2: Qualitative Coding Framework: Themes, Categories, Representative Codes, and Example Data Extracts from Conventional Content Analysis of Reflective Writings and Final Presentations (N = 26); S3: TREND Statement Checklist (Transparent Reporting of Evaluations with Nonrandomized Designs).

## Abbreviations

The following abbreviations are used in this manuscript:

CMCL	Changing Minds, Changing Lives
SDT	Self-Determination Theory
Thai CD-RISC	Thai Connor–Davidson Resilience Scale
T-PSS-10	Thai Perceived Stress Scale-10
PHQ-9	Patient Health Questionnaire-9
ANOVA	Analysis of Variance
SD	Standard Deviation
TREND	Transparent Reporting of Evaluations with Nonrandomized Designs
