# Supplementary material for: Evaluation of a Strength-Based Resilience Program on Resilience, Perceived Stress, and Depressive Symptoms Among Secondary School Students in a Thai Private School: A Single-Group Pre–Post–Follow-Up Mixed-Methods Study
Source: Healthcare (Basel). 2026 Aug 1;14(15):2337. doi: 10.3390/healthcare14152337 (PMC13464754; doi:10.3390/healthcare14152337)

Supplementary Material S1

CMCL Program: Delivery Schedule, Session Structure, and Thai-Language Class Worksheets and Homework

โปรแกรมปรับความคิด เปลี่ยนชีวิต (Changing Minds, Changing Lives)

This supplement documents the delivery schedule, session structure, and the Thai-language class worksheets and homework assignments used in the present study, to support transparency and replication. The complete CMCL facilitator manual, including full session scripts and psychoeducational content, is proprietary to the program developers (Chandler & Helling, 2022; 2Trees, LLC) and is available from them; it is therefore not reproduced here.

S1.1 Program delivery schedule

**Visit**	**Activities**
**Visit 1 (Pre-class)**	Study information and written consent/assent; online baseline assessment (T1). Duration: 1 hour.<br>*ให้ข้อมูลเกี่ยวกับการเข้าร่วมโครงการ และลงชื่อยินยอมเข้าร่วมโครงการ*<br>*รวมถึงทำแบบประเมินออนไลน์ให้เสร็จก่อนเข้าร่วมโครงการ (1 ชั่วโมง)*
**Visit 2 (Week 1)**	Sessions 1 and 2, separated by a 30-minute break. Duration: 2.5 hours.<br>*กิจกรรมที่ 1 และ 2 (2 ชั่วโมงครึ่ง) พักระหว่างกิจกรรม 30 นาที*
**Visit 3 (Week 2)**	Sessions 3 and 4, separated by a 30-minute break. Duration: 2.5 hours.<br>*กิจกรรมที่ 3 และ 4 (2 ชั่วโมงครึ่ง) พักระหว่างกิจกรรม 30 นาที*
**Visit 4 (Week 3)**	Sessions 5 and 6, followed by final group presentations and the online post-intervention assessment (T2). Duration: 3 hours.<br>*กิจกรรมที่ 5 และ 6 เข้าร่วมการนำเสนอรายบุคคลทั้งกลุ่ม และทำแบบประเมินออนไลน์หลังเข้าร่วมโครงการ (3 ชั่วโมง)*
**Follow-up (online)**	Online follow-up assessment one month after program completion (T3).<br>*ทำแบบประเมินออนไลน์หนึ่งเดือนหลังจากเข้าร่วมโครงการ (ออนไลน์)*

Note. Reflective writing was collected at the end of each session; the final group presentations in Visit 4 constituted the second qualitative data source. Sessions were scheduled for one hour each, comprising approximately 50 minutes of structured activity.

S1.2. Session-by-session structure

**Session**	**Centering practice (5 min)**	**Research presentation (15 min)**	**Writing prompt (10 min)**	**Reflection (5 min)**	**Homework**
1	Physicalize the Resilience Response	Introduction, Honing Resilience	Letter to my future self (จดหมายถึงอนาคต)<br>I am from... (ฉันมาจาก...)	AAA	Positive Practice (PP) + StrengthsQuest
2	Cloud Arms	Resilience	A time I was resilient (เมื่อไหร่กันนะที่ฉันแข็งแกร่ง)<br>I am good at (ฉันเก่งเรื่องอะไร)	AAA	PP: Gratitude + Strength
3	Body Scan	Neurobiology of Stress	A time when I or a friend felt stressed (เมื่อไหร่กันนะที่ฉัน หรือเพื่อน รู้สึกเครียด)	AAA	PP: Recover + Strength

4	Tree Pose	Stress, Health, Adversity & Resilience	Respond to objects (ความรู้สึกเมื่อเห็นสิ่งของเหล่านี้)	AAA	PP: Focus + Strength
5	Four Element	Automatic Thinking	A recent experience with automatic thinking (ความคิดอัตโนมัติทางลบ)	AAA	PP: Joy + Strength
6	Loving Kindness	Self-Compassion; Final Presentation	Goodness Cloud (เมฆแห่งความดีงาม)	AAA	Final Resilience Reflection

Note. AAA = Affirmation, Appreciation, Appraisal (closing reflection). Each session followed the five-component sequence described in Section 2.4 of the main text: centering practice (5 min), research presentation (15 min), structured reflective writing (10 min), small-group sharing with affirmative feedback (15 min), and closing AAA reflection (5 min). Homework comprised a Positive Practice exercise and identification of one personal strength; each session's worksheet began with space to record the Positive Practice and strength from the previous session's homework.

S1.3. Class worksheets and homework (as used, Thai language)

Each worksheet is reproduced below. Ruled lines for student writing are indicated as [lined space]. Positive Practice (PP) headings show the theme reviewed at the start of each session.

<u>กิจกรรมที่ 1</u> Honing Your Resilience

Writing prompt: I am from… (ฉันมาจาก…)

[lined space]

<u>กิจกรรมที่ 2</u> **Resilience**

Homework

Positive Practice (Appreciative Inquiry)

Strength 1 ข้อ

Writing prompt: A time I was resilient (เมื่อไหร่กันนะที่ฉันแข็งแกร่ง)

I am good at (ฉันเก่งเรื่องอะไร)

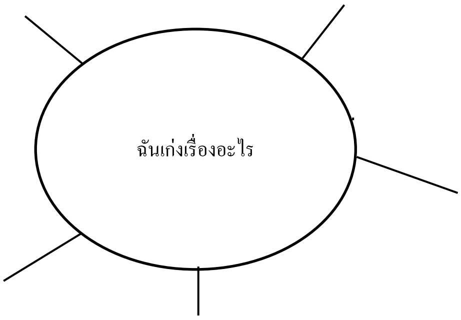

<u>กิจกรรมที่ 3</u> **Neurobiology of Stress**

<u>Homework</u>

Positive Practice (Health)

Strength 1 ข้อ

Writing prompt:
A time when you, or a friend, was stressed (เมื่อไหร่กันนะที่ฉัน หรือเพื่อน รู้สึกเครียด)

<u>กิจกรรมที่ 4</u> Stress, Health, Adversity & Resilience

<u>Homework</u>

Positive Practice (Recover)

____
____
____
____
____

Strength 1 ข้อ

____
____
____
____
____

Writing prompt: Respond to objects (ความรู้สึกเมื่อเห็นสิ่งของเหล่านี้)

____
____
____
____
____
____
____

กิจกรรมที่ 5 Automatic Thinking

Homework

Positive Practice (Focus)

Strength 1 ข้อ

Final Presentation (หัวข้อของการนำเสนอ)

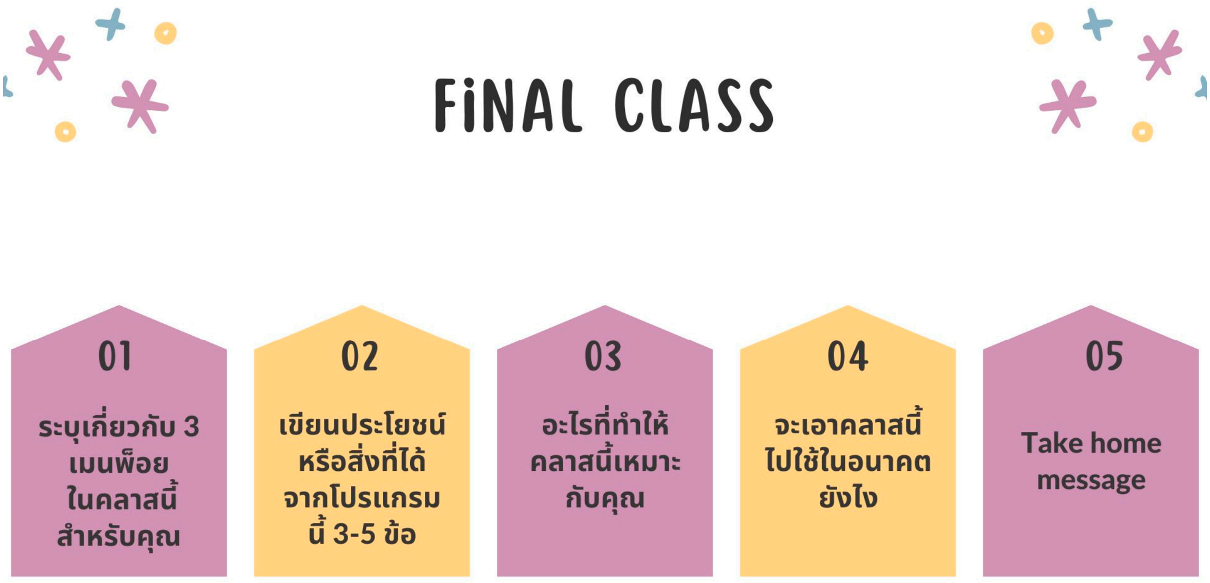

Writing prompt: Automatic Thinking Worksheet

เหตุการณ์	ความคิด, ความรู้สึก, ความรู้สึกทางกาย	ความคิดอัตโนมัติที่เกิดขึ้น	ความคิดที่มีประโยชน์ต่อใจมากกว่า
วันศุกร์ฉันไปเดินตลาดนัด เดินผ่านเพื่อนร่วมชั้น แต่เขาไม่แม้แต่ทักทาย	เพื่อนคนนั้นเขาไม่ชอบฉันแน่ ๆ ตอนแรกฉันก็โกรธนะ ต่อมาก็เริ่มรู้สึกเศร้า ห่อเหี่ยว ไหล่ตก รู้สึกไม่โอเคมากๆ	ฉันคิดว่าเขาเป็นเพื่อน แต่เขาคงไม่ชอบฉัน ฉันไม่ได้มีเพื่อนมากมาย เพราะทุกคนคงเกลียดฉัน	บางทีเขาอาจจะมองไม่เห็นฉัน เพราะคนเยอะมาก เดี๋ยวฉันลองทักแชทไปดีกว่า

กิจกรรมที่ 6 Self-Compassion

Writing prompt: Goodness Cloud (เมฆแห่งความดีงาม)

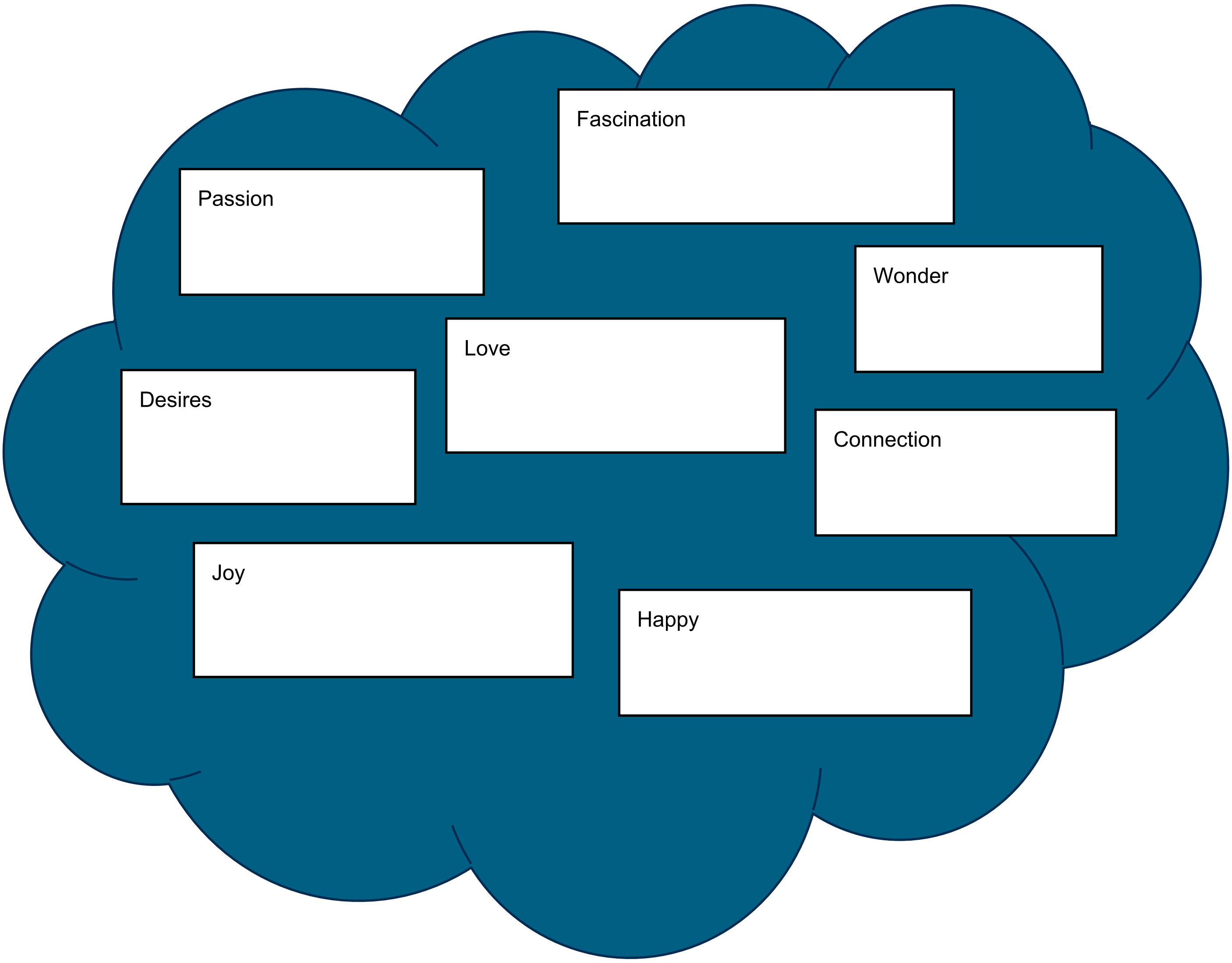

Supplement: Supplementary file 1 [file healthcare-14-02337-s001.zip › Supplementary_S1_Program_Schedule_Worksheets.pdf]
