# Supplementary material for: Evaluation of a Strength-Based Resilience Program on Resilience, Perceived Stress, and Depressive Symptoms Among Secondary School Students in a Thai Private School: A Single-Group Pre–Post–Follow-Up Mixed-Methods Study
Source: Healthcare (Basel). 2026 Aug 1;14(15):2337. doi: 10.3390/healthcare14152337 (PMC13464754; doi:10.3390/healthcare14152337)
Supplement: Supplementary file 1 [file healthcare-14-02337-s001.zip › Supplementary_S2_Coding_Framework.pdf]

Supplementary Material S2

Qualitative Coding Framework: Themes, Categories, Representative Codes, and Example Data Extracts from Conventional Content Analysis of Reflective Writings and Final Presentations (N = 26)

S2.1. Analytic approach

This supplement details the coding framework underlying the qualitative findings reported in Section 3.3 and Table 5 of the main text. All 26 participants completed a brief written reflection (approximately 100-150 words) at the end of each of the six sessions, yielding 156 reflective texts in total, with no missing reflections; the analyzed corpus comprised these 156 texts together with the 26 final presentations (one per participant, delivered to the whole group in Session 6). Analysis followed conventional (inductive) content analysis and proceeded in five stages:

- **1. Immersion.** All reflective texts and presentation records were read repeatedly to gain familiarity with the data as a whole.
- **2. Open coding.** Meaning units (words, phrases, or sentences) were coded line by line, capturing both manifest content (explicit statements) and latent content (underlying meaning).
- **3. Category formation.** Similar codes were grouped into categories reflecting shared meaning.
- **4. Theme abstraction.** Categories were abstracted into three overarching themes — Self-Understanding, Stress Management, and Understanding Others — corresponding to the program's intended domains of change.
- **5. Consensus and verification.** Two researchers coded independently; discrepancies were resolved through discussion to full consensus (a consensus-based, dialogic approach). Trustworthiness was supported by multiple-coder cross-checking, peer debriefing, and member checking, in which the preliminary themes, together with illustrative extracts, were presented to three participants, who confirmed that the themes accurately reflected their experiences; no modifications were required. Selected quotations were translated into English by the bilingual principal investigator and verified for accuracy of meaning by a second bilingual member of the research team.

Consistent with the main text, themes were derived from patterns of meaning across the dataset rather than from frequency of occurrence; participant identifiers are provided to indicate the breadth of the supporting data, not as quantitative results. "FP" denotes an extract drawn from a final group presentation; all other extracts are from reflective writings.

S2.2. Coding framework

Table S2.1 presents each theme with its definition, the representative codes reported in Table 5 of the main text (with brief descriptions), the breadth of supporting data, and illustrative extracts in the original Thai with English translations.

Theme	Definition and representative codes (Table 5)	Example data extracts (Thai original; English translation)
**Self-Understanding**	**Definition.** Recognizing one's own strengths, weaknesses, emotions, and feelings; gaining self-compassion; developing new self-perceptions; and cultivating a sense of self-worth.	"ไม่มีใครเข้าใจเรามากกว่าตัวเอง"<br>*"No one understands us better than we understand ourselves" (P01)*

Theme	Definition and representative codes (Table 5)	Example data extracts (Thai original; English translation)
	**Representative codes:**<br>• **Recognize strength and weakness** — Identifying personal strengths together with limitations, forming a balanced self-view.<br>• **Recognizing own emotions and feelings** — Noticing and naming one's own emotional states.<br>• **Gaining self-compassion** — Treating oneself with kindness rather than harsh self-criticism.<br>• **Opening a new self-perception** — Arriving at an expanded or revised view of oneself.<br>• **Having sense of self-worth** — Developing a felt sense of personal value and worthiness.<br><br>**Breadth of data.** *Present for all 26 participants when both primary and secondary coding are considered (dominant theme). Directly coded for the large majority of participants (e.g., P01, P03, P04, P08, P10, P11, P16, P17, P18, P19, P21, P24, P26).*	"การจัดการใส่ใจตัวเองให้เหมือนกับที่ใส่ใจคนที่เรารัก"<br>*"Caring for myself the way I care for someone I love" (P16)*<br><br>"หลายครั้งที่เราไม่มีเวลาคุยกับตัวเอง<br>ครั้งนี้ทำให้ได้คุยกับตัวเองมากขึ้น"<br>*"Many times we have no time to talk with ourselves; this time let me talk with myself more" (P12)*<br><br>"สามารถได้เข้าใจความรู้สึกตัวเอง"<br>*"I came to understand my own feelings" (P19)*<br><br>"ในอนาคตถ้าเรารู้สึกท้อ<br>เราจะลองมองตัวเองให้ละเอียดมากขึ้น"<br>*"In the future, if I feel discouraged, I will try to look at myself more carefully" (P26)*
**Stress Management**	**Definition.** Alleviating negative emotions through modified coping strategies, positive thinking, assertiveness development, problem-solving approaches, and venting negative feelings with friends.<br><br>**Representative codes:**<br>• **Learning and modified various ways of coping methods** — Acquiring and adapting coping strategies, often through peer observation.<br>• **Positive thinking** — Adopting more optimistic or balanced appraisals of stressful situations.<br>• **Assertiveness** — Expressing needs and setting boundaries more confidently.<br>• **Problem-solving approach** — Analysing problems and identifying constructive courses of action.<br>• **Venting negative feelings** — Releasing or expressing distress, including with peers, for relief.<br><br>**Breadth of data.** *Coded for 14 participants (e.g., P02, P03, P06, P07, P08, P10, P12, P13, P17, P20, P21, P22, P25, P26).*	"ได้รู้วิธีกำจัดความเครียดของตัวเองและนำของเพื่อนมาปรับใช้"<br>*"I learned my own ways of relieving stress and adapted my friends' ways to myself" (P02)*<br><br>"สิ่งที่อุดอู้ในใจมันเริ่มเบาลงบ้าง"<br>*"The things bottled up inside began to feel lighter" (P21)*<br><br>"ทำให้อาการเครียดจากงานโปรเจคน้อยลง"<br>*"My stress from project work has decreased" (P25)*<br><br>"เวลาเครียดเราจะคิดในแง่บวกมากขึ้น"<br>*"When stressed, I now think more positively" (P07)*<br><br>"เรามีวิธีมองปัญหาและรู้ว่าจะจัดการกับมันยังไง"<br>*"I have a way of seeing my problem and know how to deal with it" (P14)*

Theme	Definition and representative codes (Table 5)	Example data extracts (Thai original; English translation)
**Understanding Others**	**Definition.** Cultivating careful, empathetic listening and gaining insight into others' motivations, perspectives, and behaviour without judgement.<br><br>**Representative codes:**<br>• **Empathetic listening to others —** Listening attentively and without judgement to understand others' experiences.<br>• **Gaining understanding of others' view —** Taking others' perspectives and appreciating differing viewpoints.<br><br>**Breadth of data.** *Coded for 13 participants (e.g., P01, P02, P03, P05, P06, P09, P11, P13, P14, P15, P18, P21, P26); related interpersonal connection and empathy were expressed by additional participants.*	"จะใช้ทักษะการรับฟังผู้อื่นและการเข้าใจผู้อื่นโดยไม่ตัดสิน"<br>*"I will use the skills of listening to others and understanding others without judging" (P05)*<br><br>"เราไม่โดดเดี่ยวแต่มีคนอยู่ด้วยเสมอ"<br>*"We are not alone; there is always someone with us" (P04)*<br><br>"ได้รับฟังมุมมองต่างของเพื่อน"<br>*"I listened to my friends' different perspectives" (P15)*<br><br>"คิดถึงใจเขาใจเรา"<br>*"Think of their feelings as we would our own [putting oneself in another's position]" (P09)*

Note. Themes and representative codes correspond exactly to Table 5 of the main text. Descriptions of codes are provided here to aid interpretation. Participant identifiers (e.g., P01) denote individual participants; FP marks a final-presentation extract.

S2.3. Additional analytic observations

Theme prevalence and interrelation

Self-Understanding was the dominant and most pervasive theme, frequently co-occurring with the other two: participants' growing self-knowledge appeared to underpin both their stress-management strategies and their understanding of others. Stress Management was the second most represented theme and was most directly linked to the program's coping content, while Understanding Others captured the interpersonal and empathic growth fostered by the small-group sharing structure.

Divergent (negative) cases

Two participants' accounts diverged from the dominant patterns and were noted as an incidental observation during analysis (not a pre-specified analytic step). P13 described emotion-regulation strategies as "rarely applied" in daily life before the program, and P22 described himself as someone who had "paid little attention to managing stress" beforehand. Both nevertheless engaged meaningfully with the program, suggesting it may be accessible to adolescents with lower baseline coping readiness (see Section 4.1 of the main text).

Higher-baseline cases

Two participants appeared to enter with higher baselines: P05 reported a pre-existing interest in studying psychology (higher baseline empathy and active listening), and P20 reported that a growth-mindset approach was already practised in his family before the program.

Transfer of learning

Several participants reported applying program content beyond the classroom: use of coping strategies at home (P03), greater willingness and confidence to talk with parents (P23), an intention to pass the skills on to future children (P11), and preparation for future working life (P18). These accounts indicate transfer of learning to family and future-oriented contexts.

Facilitator influence

One participant (P06) directly credited the facilitator's accessible communication style and engaging materials with enabling his participation, an observation relevant to implementation quality as a moderator of outcomes.
