# Supplementary material for: Evaluation of a Strength-Based Resilience Program on Resilience, Perceived Stress, and Depressive Symptoms Among Secondary School Students in a Thai Private School: A Single-Group Pre–Post–Follow-Up Mixed-Methods Study
Source: Healthcare (Basel). 2026 Aug 1;14(15):2337. doi: 10.3390/healthcare14152337 (PMC13464754; doi:10.3390/healthcare14152337)
Supplement: Supplementary file 1 [file healthcare-14-02337-s001.zip › Supplementary_S3_TREND_Checklist.pdf]

Supplementary Material S3

TREND Statement Checklist (Transparent Reporting of Evaluations with Nonrandomized Designs)

The checklist follows the TREND statement (Des Jarlais, Lyles, Crepaz, & the TREND Group, 2004) [16]. Item descriptors are condensed from the original checklist. "Not applicable" indicates items that do not apply to a single-group design without a comparison condition. All section, table, and figure numbers refer to the revised manuscript.

Item	Paper section / topic	Descriptor (condensed)	Reported in
1	**Title and Abstract**	Information on how units were allocated to interventions; structured abstract; information on target population/study sample	Title; Abstract (single-group design and absence of a control group stated; sample described as 26 students in a single-sex boys' secondary school)
2	**Introduction: Background**	Scientific background, rationale, and theories used in designing the intervention	Section 1 (Self-Determination Theory; ABCs of resilience framework; rationale and study gaps)
3	**Methods: Participants**	Eligibility criteria; recruitment method; recruitment setting; settings of data collection	Section 2.2 (eligibility criteria, recruitment procedure, enrollment cap, setting); Section 2.1 (study setting and period); Figure 1
4	**Methods: Interventions**	Details of the intervention: content, delivery method, unit of delivery, deliverer, setting, exposure quantity and duration, activities to increase or assess fidelity	Section 2.4 (TIDieR-informed description: content, five-component session sequence, facilitators, eight hours of facilitator training, delivery schedule, fidelity monitoring); Section 2.5 (translation and cultural adaptation); Table 1; Supplementary S1
5	**Methods: Objectives**	Specific objectives and hypotheses	Section 1, final paragraph (two explicit objectives: quantitative and qualitative)
6	**Methods: Outcomes**	Clearly defined primary and secondary outcome measures; instrument validity and reliability	Section 2.6 (Thai CD-RISC, T-PSS-10, PHQ-9; administration at T1, T2, T3); Section 2.7 (Cronbach's alpha at baseline in the present sample)
7	**Methods: Sample size**	How sample size was determined	Section 2.2 (a priori G*Power calculation for repeated measures; explicit statement that it does not support causal inference; justification of qualitative data adequacy)
8	**Methods: Assignment method**	Unit of assignment; method of assignment; restrictions	Not applicable — single-group design with no allocation or comparison group (Section 2.1)
9	**Methods: Blinding (masking)**	Whether participants, deliverers, or assessors were blinded	Blinding not performed; outcomes were self-reported and facilitators were members of the research team; acknowledged as a limitation (Sections 2.9 and 4.3)
10	**Methods: Unit of analysis**	Description of the smallest unit analyzed; whether it matches the unit of assignment	Section 2.8 (individual participant as the unit of analysis)

Item	Paper section / topic	Descriptor (condensed)	Reported in
11	**Methods: Statistical methods**	Statistical methods for primary outcomes; additional analyses; methods for missing data; software	Section 2.8 (repeated measures ANOVA; Shapiro-Wilk and Mauchly tests; Greenhouse-Geisser correction; Bonferroni-corrected post hoc comparisons with adjusted 95% confidence intervals; participant-level mean imputation for minimal item-level missing data; rationale for retaining ANOVA over a linear mixed model; IBM SPSS Statistics 31.0); Section 2.9 (conventional content analysis; consensus-based coding); Section 2.10 (convergent mixed-methods integration)
12	**Results: Participant flow**	Flow of participants through each stage (enrollment, assignment, allocation, follow-up, analysis), ideally with a diagram	Figure 1 (TREND-style flow diagram); Sections 2.2 and 3.1 (26 volunteers, none excluded, all 26 attended all six sessions and completed T1-T3; zero attrition)
13	**Results: Recruitment**	Dates defining periods of recruitment and follow-up	Section 2.1 (study conducted between November 2025 and March 2026, including one-month follow-up)
14	**Results: Baseline data**	Baseline demographic and clinical characteristics	Table 2 (participant characteristics); Section 3.1 (baseline PHQ-9 screening; no participant scored 15 or above or endorsed item 9)
15	**Results: Baseline equivalence**	Baseline comparisons of study groups	Not applicable — single-group design with no comparison group
16	**Results: Numbers analyzed**	Number of participants included in each analysis; whether analysis was by original assignment	Section 3.1 and Figure 1 (N = 26 in all quantitative analyses; complete data at all three time points); Section 2.9 (qualitative corpus of 156 reflective texts, all 26 participants across six sessions with no missing reflections, plus 26 final presentations)
17	**Results: Outcomes and estimation**	Summary of results for each outcome, including effect sizes and confidence intervals	Section 3.2; Table 3 (means, SD, F, df, partial eta squared); Table 4 (mean differences, SE, Bonferroni-corrected p values, Bonferroni-adjusted 95% CIs); Figure 2
18	**Results: Ancillary analyses**	Other analyses performed, distinguishing pre-specified from exploratory	Section 3.3 (qualitative themes); Section 3.4 and Table 6 (mixed-methods joint display); Section 4.1 (divergent cases reported explicitly as an incidental, non-pre-specified observation)
19	**Results: Adverse events**	All important adverse events or unintended effects	Section 2.3 (pre-specified safety protocol, risk assessment and referral pathway); Section 3.1 (no participant required activation of the protocol; no adverse events observed)
20	**Discussion: Interpretation**	Interpretation considering hypotheses, sources of bias, imprecision, and multiplicity	Section 4.1 (interpretation of each outcome with design limitations integrated; associative rather than

Item	Paper section / topic	Descriptor (condensed)	Reported in
			causal language); Section 4.3 (absence of a control group, possible inflation of effect sizes, social desirability, measurement limitations)
**21**	**Discussion: Generalizability**	Generalizability (external validity) of findings	Section 4.3 (single private all-boys school in Bangkok; small homogeneous sample; limits to generalizability to female students, public schools, and rural or lower socioeconomic settings)
**22**	**Discussion: Overall evidence**	General interpretation of results in the context of current evidence	Sections 4.1 and 4.2 (findings situated within prior resilience-intervention literature; implications for school practice); Section 5 (preliminary findings; controlled studies required before broader implementation)

Reference: Des Jarlais, D.C.; Lyles, C.; Crepaz, N.; TREND Group. Improving the reporting quality of nonrandomized evaluations of behavioral and public health interventions: The TREND statement. *Am. J. Public Health* **2004**, *94*, 361–366. https://doi.org/10.2105/AJPH.94.3.361
